# A Qualitative Systematic Review of Facilitators of and Barriers to Community Pharmacists–Led Anticoagulation Management Service

**DOI:** 10.1177/10600280211045075

**Published:** 2021-09-11

**Authors:** Oluwaseun Egunsola, Joyce W. Li, Liza Mastikhina, Oluwasefunmi Akeju, Laura E. Dowsett, Fiona Clement

**Affiliations:** 1 University of Calgary, Calgary, Alberta, Canada

**Keywords:** pharmacy, anticoagulation, warfarin, community

## Abstract

**Objective:**

To identify the facilitators of and barriers to the implementation of Community Pharmacists–Led Anticoagulation Management Services (CPAMS).

**Data Sources:**

MEDLINE, EMBASE, CINAHL, Cochrane Database of Systematic Reviews, and Cochrane CENTRAL Register of Controlled Trials were searched from inception until August 20, 2021.

**Study Selection and Data Extraction:**

All abstracts proceeded to full-text review, which was completed by 2 reviewers. Data extraction was completed by a single reviewer and verified. Analysis was completed using best-fit framework synthesis.

**Data Synthesis:**

A total of 17 articles reporting on CPAMS from 6 jurisdictions were included: 2 Canadian provincial programs (Nova Scotia, Alberta), a national program (New Zealand), and 3 cities in the United Kingdom (Whittington and Brighton and Hove) and Australia (Sydney). Facilitators of CPAMS included convenience for patients, accessibility for patients, professional satisfaction for pharmacists, increased efficiency in anticoagulation management, improved outcomes, enhanced collaboration, and scalability. Barriers included perceived poor quality of care by patients, resistance by general practitioners, organizational limits, capping of the number of eligible patients, and cost.

**Relevance to Patient Care and Clinical Practice:**

The barriers and facilitators identified in this review will inform health policy makers on the implementation and improvement of CPAMS for patients and health care practitioners.

**Conclusion and Relevance:**

CPAMS has been implemented in 6 jurisdictions across 4 countries, with reported benefits and challenges. The programs were structurally similar in most jurisdictions, with minor variations in implementation. New anticoagulation management programs should consider adapting existing frameworks to local needs.

## Background

Warfarin has been available and used for the prevention and treatment of thromboembolic events for more than 70 years.^1^ It has been shown to reduce the risk of stroke in patients with atrial fibrillation by 60%.^1^ To better manage warfarin therapy, some jurisdictions have established specialized anticoagulation clinics.^1,2^ These anticoagulation management services (AMSs) are provided by health care professionals such as doctors, nurses, and pharmacists, independently or collaboratively.

Anticoagulation services were traditionally provided by physicians in collaboration with onsite or off-site laboratories.^3^ However, patient-centered models, such as self-testing and self-management, are emerging alternatives that allow those on warfarin to be more involved with their own care. In the self-testing model, the patients perform international normalized ratio (INR) testing by themselves with a point-of-care device and only contact health professionals for interpretation and dose adjustment.^4^ The self-management model requires patients to monitor their INR values directly, interpret the results, and adjust their warfarin doses using a dosing algorithm.^5^ Other jurisdictions utilize multidisciplinary approaches to anticoagulation management, with differing levels of responsibility for the members of the health care team.^2^ Examples of multidisciplinary AMS models include pharmacists-led,^6,7^ pharmacists-assisted,^8,9^ and nurse-led^10^ care models. Pharmacists-led anticoagulation services can be provided at the local pharmacies by community pharmacists or in-hospital by clinical or any licensed pharmacists.^11,12^

Because community pharmacists are the most accessible of all health care providers,^13^ their involvement in anticoagulation management clinics could significantly increase patients’ access to AMSs.^14^ Community Pharmacists–Led Anticoagulation Management Services (CPAMS) entail the provision of INR point-of-care testing by community pharmacists and the adjustment of warfarin dose in line with an approved decision-support system.^15^

Despite its potential for improving anticoagulation management, CPAMS has not been widely adopted. Therefore, the purpose of this systematic review is to identify the facilitators of and the barriers to the implementation of CPAMS across different jurisdictions.

## Methods

### Search Strategy

A systematic review of the literature was completed. Literature search was conducted in accordance with the Preferred Reporting Items for Systematic reviews and Meta-analyses literature search extension guideline.^16^ A protocol was not registered. MEDLINE, EMBASE, CINAHL, Cochrane Database of Systematic Reviews, and Cochrane CENTRAL Register of Controlled Trials were searched from inception until August 20, 2021. The search strategy was developed by a research librarian, and peer-review of electronic search strategies was completed by another research librarian.^17^ The full search strategy is reported in the appendix. The database search was supplemented by a gray literature search guided by the Canadian Agency for Drugs and Technologies in Health’s (CADTH) “Grey Matters” document^18^ and Google search.

### Literature Selection

Calibration with a second reviewer was completed prior to abstract screening and full-text review until >70% agreement was reached. Abstracts identified through database searching were screened in duplicate; all abstracts included by either reviewer proceeded to full-text review. Full-text publications were screened in duplicate. Although the intervention of a third reviewer was planned for any discrepancy, this was not necessary because all discrepancies were resolved by discussion between the 2 reviewers until a consensus was reached. This typically relied on the ability of each reviewer to justify the inclusion or exclusion of the studies. Publications were excluded if they did not meet the inclusion criteria or if the study was not available in English or French. Inclusion criteria were studies or reports on CPAMS program implementation, qualitative evaluation of CPAMS, and program overview. A variety of study designs was expected; therefore, no risk-of-bias assessment was planned.

### Data Extraction and Synthesis

A best-fit framework synthesis methodology was used as described by Carroll et al.^19^ This involved the development of an a priori framework, which provided a structure to the coding and analysis. The framework was developed and translated into nodes and subnodes in NVivo qualitative data analysis software; QSR International Pty Ltd, Version 12. Each subnode represented facilitators of and barriers to CPAMS identified during full-text screening. To ensure consistency between the 2 reviewers, pilot coding was conducted and areas of disagreement discussed. Participant quotations and author synthesis were coded.

Data on study characteristics were extracted onto an Excel sheet by a primary reviewer while another reviewer verified. Disagreements were resolved by discussion. Extracted data included author name, country of study, year of study, type of publication, number of participants, and participant’s demographic characteristics.

Broadly, data synthesis involved the following: (1) description of the review question, (2) identification of relevant literature, (3) framework development, (4) coding the relevant evidence, (5) creation of new themes for those data not captured by the a priori framework, (6) development of an updated framework, (7) understanding the relationships between themes, and (8) narratively describing the findings under 2 broad themes—an overview of CPAMS program implementation and facilitators of and barriers to CPAMS.

This study was conducted in accordance with the Enhancing Transparency in Reporting the Synthesis of Qualitative Research statement.^20^

## Results

### Characteristics of Included Studies

**Figure 1.**
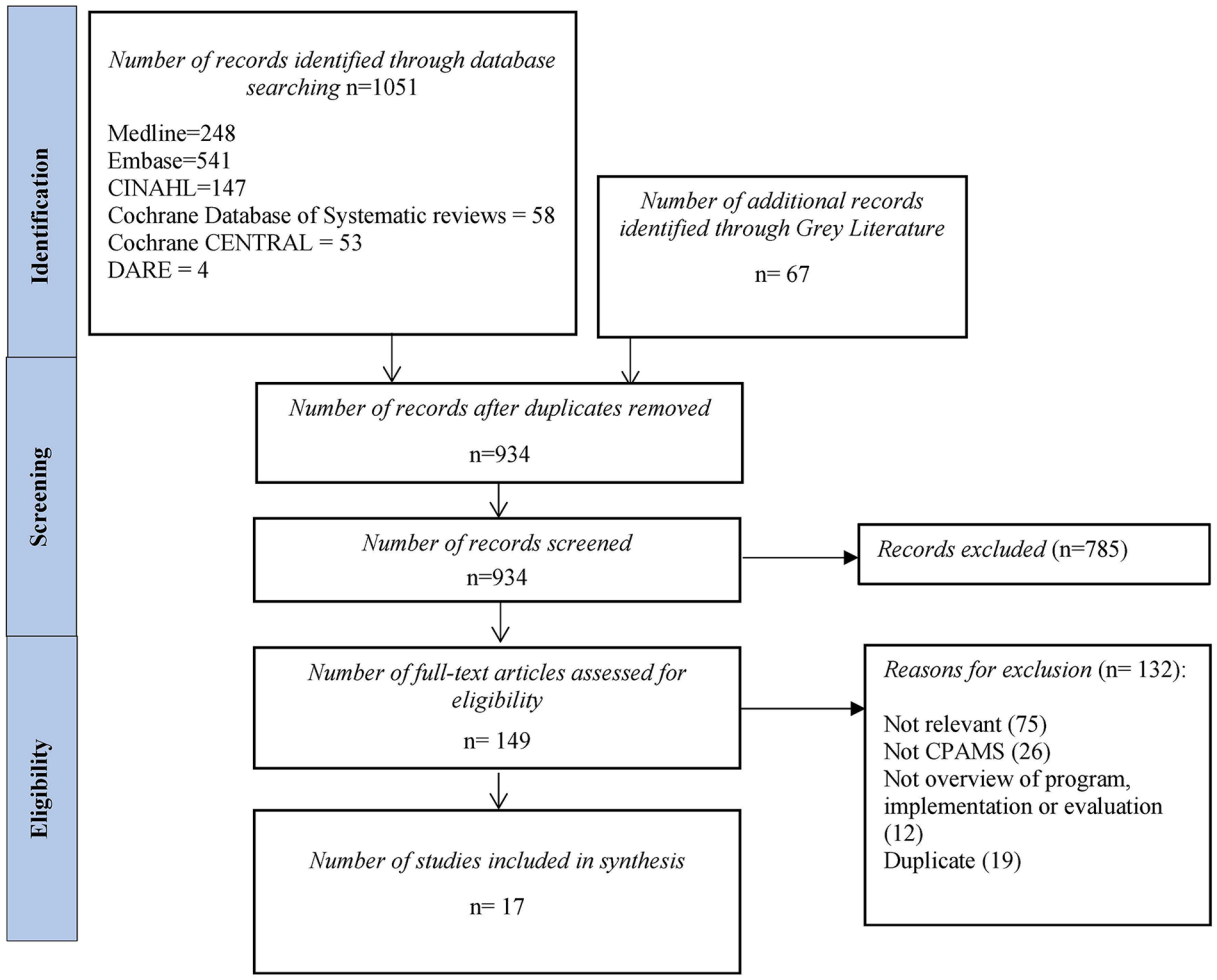
PRISMA flowchart of included studies. Abbreviation: CPAMS, Community Pharmacists–Led Anticoagulation Management Services.

The search strategy yielded 934 unique citations. After abstract screening, 149 studies proceeded to full-text review (Figure 1). A total of 133 studies were excluded for the following reasons: not relevant (n = 75), not CPAMS (n = 26), duplicate (n = 19), and did not provide an overview of program, implementation or evaluation (n = 12; Figure 1). In all, 17 studies^15,21-36^ were included in the final narrative synthesis; 9 of the included studies reported on New Zealand’s CPAMS,^27-35^ 3 reported on Nova Scotia’s (Canada) program,^15,21,26^ 2 involved Boots, the UK anticoagulation program,^22,25^ and 1 each involved the Alberta (Canada),^23^ Whittington (UK),^24^ and Sydney (Australia)^36^ programs (Table 1).

**Table 1.** Table of Study Characteristics.

Country	Author	Year	Type of publication	Study design	Number of Participants	Participant characteristics	Testing device
UK	Ingram et al^22^	Publication year: 2019 Implementation year: 2009	Journal article	Mixed method	Pharmacies: 8 Pharmacists: 7 Patients: 2341	Age: 22-106 years Percentage female: 44%	CoaguChek XS Plus
	Coleman et al^24^	Publication year: 2003 Implementation year: 2002	Journal article	Report	Pharmacies: 1 Pharmacists: 2 Patients: 18	Age: NR Percentage female: NR	NR
	Royal Pharmaceutical Society^25^	Publication year: 2016 Implementation year: 2016	Electronic report	Report	Pharmacies: NR Pharmacists: NR Patients: 80	Age: NR Percentage female: NR	NR
Canada	Bungard et al^23^	Publication year: 2006 Implementation year: NR	Journal article	Report	Pharmacies: 7 Pharmacists: 14 Patients:	Age: NR Percentage female: NR	Laboratory
	Woodill and Bodnar^21^	Publication year: 2020 Implementation year: 2018	Journal article	Report	Pharmacies: 40 Pharmacists: Patients: 946	Age: NR Percentage female: NR	CoaguChek XS Plus
	Pharmacy Association of Nova Scotia^26^	Publication year: 2019 Implementation year: 2018	Electronic report	NA	Pharmacies: 40 Pharmacists: 106 Patients: 946	Age: NR Percentage female: 56.6%	Coaguchek XS Pro
	Pharmacist Association of Nova Scotia^15^	Publication year: NR Implementation year: NR	Web article	NA	Pharmacies: 40 Pharmacists: Patients:	Age: NR Percentage female: NR	CoaguChek XS Pro
New Zealand	Community Pharmacy Warfarin Service^31^	Publication year: 2011 Implementation year: 2012-2013	Report	NA	Pharmacies: 125 Pharmacists: NR Patients: NR	Age: NR Percentage female: NR	CoaguChek XS Plus device
	Harrison et al^28^	Publication year: 2015 Implementation year: 2010-2011	Journal article	Prospective cohort	Pharmacies: NR Pharmacists: 41 Patients: 693	Age: 72 years, median (13-97) Percentage female: 37.6	CoaguChek XS Plus device
	Shaw et al^29^	Publication year: 2014 Implementation year: NA	Journal article	Mixed methods	Pharmacies: 15 Pharmacists: NR Patients: 693	Age: NR Percentage female: 37.6	CoaguChekXS Plus
	Harper et al^30^	Publication year: 2015 Implementation year: 2013-2014	Journal article	Retrospective cohort	Pharmacies:126 Pharmacists: NR Patients: 5866	Age: 67.65 years, mean Percentage female: NR	NR
	Shaw et al^32^	Publication year: 2016 Implementation year: 2010-2011	Journal article	Mixed methods	Pharmacies: NR Pharmacists: 41 Patients: 693	Age: NR Percentage female: 37.6	CoaguChekXS Plus
	Beyene et al^33^	Publication year: 2020 Implementation year: 2019	Journal article	Cross-sectional	Pharmacies: 33 Pharmacists: Patients: 305	Age: NR Percentage female: 43.3	NR
	Beyene et al^34^	Publication year: 2020 Implementation year: 2018	Journal article	Mixed methods	Pharmacies: 133 Pharmacists: Patients: NR	Age: NR Percentage female: 44.1	NR
	National CPAMS Quality Report^35^	Publication year: 2016 Implementation year: NR	Report (information sheet)	NA	Pharmacies: 150+ Pharmacists: NR Patients: 6000+	Age: NR Percentage female: NR	NR
	Shaw et al^27^	Publication year: 2011 Implementation year: 2010-2011	Report	Report	Pharmacies: 15 Pharmacists: 41 Patients: 693	Age: 72 years, median (19-97) Percentage female: 37.6	CoaguChekXS Plus
Australia	McLachlan et al^36^	Publication year: 2005 Implementation year: 2004	Electronic report	Report	Pharmacies: 7 Pharmacists: Patients: 44	Age: 71.9 ± 1.6 years Percentage female: 25%	INRatio

Abbreviations: NA, not applicable; NR, not reported.

Nine of the studies were program descriptions of CPAMS.^15,21,23-27,31,35^ There were 5 mixed-method articles. These included the following: a survey and interview of pharmacists on their experience of CPAMS^34^; a survey and interview of patients, general practitioners (GPs), practice nurses, and pharmacists^29^; an economic evaluation and cross-sectional survey of patients and health care workers^32^; a prospective controlled study of the effectiveness of CPAMS, an economic evaluation, and a semistructured interview^36^; and a database analysis and survey of experiences of patients attending CPAMS.^22^ There was 1 cross-sectional survey of patients in the New Zealand CPAMS program that evaluated their satisfaction with the program,^33^ 1 clinical audit of patients to determine anticoagulant control and compliance with CPAMS,^30^ and a prospective cohort study comparing effectiveness of CPAMS with GP-led care.^28^

#### Overview of CPAMS Implementation Across Jurisdictions

Across jurisdictions, patients were eligible to enroll in a CPAMS program after referral from their physicians. These were generally medically stable patients without significant comorbid conditions. The community pharmacists were empowered to prescribe warfarin, monitor INR with a point-of-care device, and adjust warfarin dosing. Whereas the Canadian and New Zealand programs were primarily driven by provincial and national health agencies, respectively, the Brighton and Hove program in the United Kingdom was facilitated by a pharmacy chain in collaboration with independent pharmacies.^22^ In most jurisdictions, community pharmacies volunteered to participate; however, in the Alberta program, pharmacists were required to volunteer and encouraged to establish CPAMS at their local community or hospital pharmacies.^23^ Furthermore, the Alberta program allowed some flexibility on program implementation, depending on the location of the pharmacies and availability of resources.

**Figure 2.**
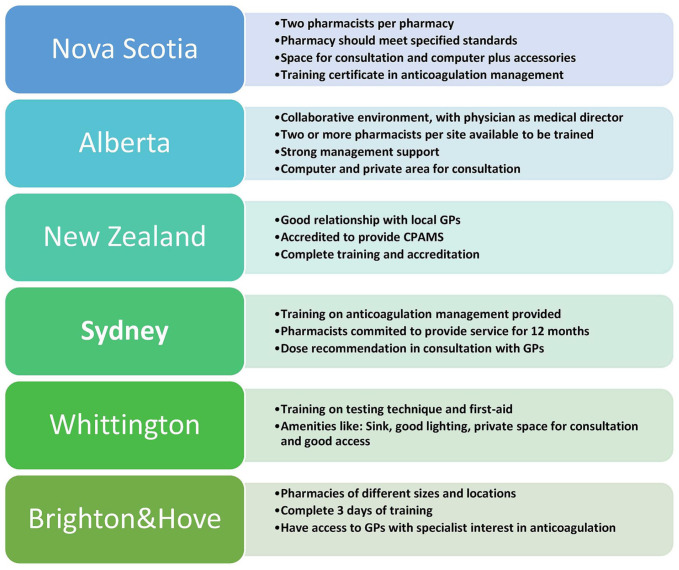
CPAMS program highlight across jurisdictions. Abbreviations: CPAMS, Community Pharmacists–Led Anticoagulation Management Services; GP, general practitioner.

Across jurisdictions, training on anticoagulation management was facilitated by an academic institution. The New Zealand and Nova Scotia programs utilized the INR Online decision support system; while the Brighton and Hove program utilized the DAWN system.^22^ In all jurisdictions, physicians continued to provide clinical support for difficult and complicated cases through established communication channels. A brief overview of each jurisdiction is provided in the next section and described in Figure 2.

#### CPAMS in Nova Scotia, Canada

In all, 40 of the 75 pharmacies that applied for the CPAMS demonstration project in Nova Scotia were selected to participate. The selection was implemented such that all counties within the province and all pharmacy banners or chains were represented.^21^ Each participating location selected a minimum of 2 pharmacists,^21^ with a total of 106 pharmacists participating in the program. Participating pharmacies were paid an INR management fee of $50 CAD per month per patient.^26^ Patients were referred by their GPs, who were provided with a referral template, which included details of a collaborative management plan.^15^ Supplementary Figure 1 (available online) describes the structure of the CPAMS program in Nova Scotia.

#### CPAMS in Alberta, Canada

In Alberta, anticoagulant management services were implemented using a 3-staged approach.^23^ The first stage involved the establishment of an AMS within a quaternary care setting; the second involved the training of selected pharmacists; and the third involved the implementation of community AMSs.^23^ A total of 7 pharmacies and 14 pharmacists were involved in this program. All program sites were required to have a minimum of 1 physician serving as the medical director, and pharmacists were required to commit to undergo the requisite training and hold anticoagulation clinics 3 days per week.^23^

#### CPAMS Implementation in New Zealand

The first pilot CPAMS project at a community pharmacy in New Zealand was implemented in 2009, followed by a larger pilot in late 2010.^27^ The participating GPs transferred authorities for specific aspects of anticoagulation management, for specific patients to the pharmacists, using informed consent forms and standing order delegations.^29,35^ Eligibility for referral to the program was dependent on the absence of specific comorbidities.^28^ Pharmacies were required to have preexisting good relationships with local GPs. All participating pharmacists were paid per patient to cover the cost of time and consumables and to set up the program.^28^

#### CPAMS in Brighton and Hove, UK

In 2009, a UK pharmacy chain began to operate a community pharmacist–led anticoagulation service in the Brighton and Hove area of England.^22^ A total of 17 pharmacies participated in the program.^22^ All pharmacists completed a 3-day training course on anticoagulation management at the National Centre for Anticoagulation and also received training on service procedures.^22^

#### CPAMS in Whittington, UK

In 2002, a 9-month pilot community pharmacy–based AMS was implemented at a community pharmacy close to Whittington, UK.^24^ Pharmacists received training on the finger-prick technique for blood sampling, use of a portable coagulometer, and use of the computerized anticoagulant advisory system. They also attended a 1-day first-aid course.^24^ This was an appointment-based service, which operated 1 afternoon every week and was managed by 2 pharmacists.^24^ An audit system was implemented to ensure the accuracy of the coagulometer.^24^

#### CPAMS in Sydney, Australia

In 2004, pharmacists from 8 community pharmacies in the Sydney metropolitan area were invited to participate in a CPAMS program in order to compare clinical outcomes with usual care provided in 5 other pharmacies.^36^ Interested pharmacists attended an educational seminar on anticoagulation management and committed to providing the service to patients participating in the study for 12 months.^36^ The pharmacists provided consultation at least once per month to check the patients’ INR, provide dietary and lifestyle advice, make dosing recommendations, and monitor adherence to anticoagulant treatment.^36^ The dosage recommendations were made in consultation with the patients’ GPs, and no dosing decisions were made without the final approval of the GPs.^36^

### Facilitators of CPAMS Across Jurisdictions (Figure 3)

**Figure 3.**
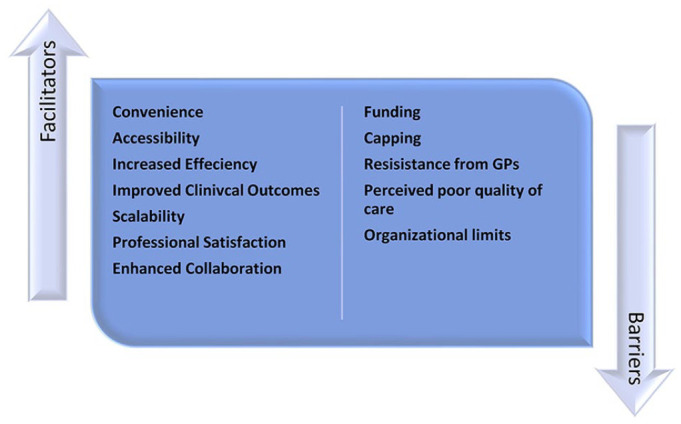
Facilitators of and barriers to CPAMS. Abbreviations: CPAMS, Community Pharmacists–Led Anticoagulation Management Services; GP, general practitioner.

#### Convenience

Patient participants reported that CPAMS was more convenient than usual GP care.^21,36^ According to a patient in the Sydney program, “It was quick and easy, no waiting and no booking”^36^

Much of the time gained was from the point-of-care testing, for which the results were available almost immediately.^21^ Several patients reported that it was easy for them to go for their anticoagulation treatment at the same time as they went for their local shopping, and some pharmacies offered flexibility in appointment, which were not available with GPs.^29^ One convenience noted by GPs was that they were able to reallocate the extra time and resources to other areas of their practice.^15^

In addition, capillary samples for point-of-care testing were easier to collect than venous samples for laboratory tests.^21^ The immediate availability of INR results, immediate dose adjustments, and a reduction in waiting time also contributed to lower stress reported across patients.^26^

“I am pleased to have the results of my reading immediately and that they prick my finger not my arm.”^26^

#### Accessibility

Some patients found pharmacists to be easily accessible. As a result, they were able to confer with them about issues they believed did not necessitate a GP consultation. This resulted in a better understanding of their condition.^29^ Some patients found the hospitals to be too busy but were able to easily get appointments with their pharmacists under the CPAMS program.^26^ The ease of securing an appointment resulted in an increase in the number of scheduled appointments for some patients.^26^

#### Increased Efficiency for the Patient and System

Several patients found CPAMS to be more streamlined than GP care, given that all aspects of anticoagulation management could be carried out in the pharmacy.^27^ CPAMS allowed some of the GPs to spend less time on warfarin management, particularly on reviewing results and adjusting doses. The GPs reported being pleased that the pharmacists were handling these aspects of their patient care.^27^ It was also reported to be easier for the pharmacists to modify warfarin doses at the time of consultation without having to rely on the patient’s laboratory bloodwork results.^21^ The provision of information in real time also meant that the GPs did not have to track down patients to relay test results and dosing information.^21^

According to one patient, “The doctor’s system is in four stages . . . blood taken, to lab, lab faxes to doctor’s reception, to duty nurse, then doctor approves. Too many rooms for mistakes.”^27^

Some participants highlighted the ability of the CPAMS model to improve efficiency in the health care system. According to them, CPAMS has the potential to reduce the burden on GPs and laboratories.^32^ GPs in New Zealand were able to reallocate their time and resources to other aspects of their practice, which resulted in overall time and cost savings.^32^ The cost of delivering CPAMS in New Zealand was 30% lower than the cost of the standard care model.^32^ The program was also believed to have kept patients out of the emergency room because of enhanced access to care.^26^ CPAMS freed up capacity in the hematology departments of some hospitals in the United Kingdom, allowing increased focus on patients with more complex needs.^24^

#### Improved Clinical Outcomes

CPAMS in Nova Scotia resulted in increased adherence to testing and medications, with almost 80% of INR tests completed on or before their due date. Patients generally found the dosing schedule provided by community pharmacists to be easier than usual care.^26^ In addition, health outcomes, such as time within therapeutic range, were reportedly better with CPAMS in Nova Scotia than usual care.^26^ Approximately 71% of patients in the CPAMS program had INR in therapeutic range compared with historical values of 50% to 60%.^26^ Participants felt that CPAMS increased the safety of anticoagulation management because INR results and dose adjustments were immediately available.^26^

#### Scalability

CPAMS was implemented in several jurisdictions as pilot programs, involving limited numbers of patients and pharmacies. Some of the interviewed pharmacists believed that it would be easy to implement the program on a larger scale as soon as the constraints of lack of funding and trained pharmacies were addressed.^26^

#### Professional Satisfaction for Pharmacists

Pharmacists across several jurisdictions identified professional satisfaction as one of the reasons for participating in the program.^21,24,27,36^ Some pharmacists in New Zealand had thought that their clinical skills were underutilized prior to CPAMS and found the program to be a natural extension of the counseling and dispensing they had been providing to patients receiving anticoagulants.^27^ For some pharmacists in the United Kingdom, it was an opportunity for clinical development and practice toward being an independent prescriber.^24^ In Nova Scotia, some pharmacists reported improved job satisfaction, finding it rewarding to use their clinical skills to work more closely with patients.^21^

“CPAMS is the most rewarding project I have been involved in as a pharmacist thus far in my career. I truly enjoyed the collaboration, the patient experiences, and the [opportunity] to practice at a higher level. I always see the benefits to our patients and the cost savings to our health care system. It’s a win for all.”^26^

For several pharmacists participating in the Nova Scotia and New Zealand CPAMS programs, their comfort and confidence in managing anticoagulation increased substantially over the course of the program.^26,27^ This increase in clinical confidence among New Zealand pharmacists led to the implementation of additional clinical services, such as medicines use review and smoking cessation support.^27^

#### Enhanced Collaboration

Some patients noted that CPAMS fostered collaboration between them, the GPs, and pharmacists.^36^

“There was a three-way communication between me, the pharmacist and the doctor”^36^

Although several pharmacists already had good working relationships with GPs, CPAMS strengthened many of these existing relationships.^26^ GPs and patients also developed a better understanding of the value that pharmacists provide to patients.^21^ The interaction between patients and pharmacists increased patients’ involvement with their treatment, which contributed to improved adherence.^27^ Pharmacists also noted that patients’ trust and confidence in them increased over time and they were more likely to share relevant health information with them. As a result, the pharmacists were able to identify other health needs and address them.^26^

### Barriers to CPAMS Across Jurisdictions (Figure 3)

#### Perceived Quality of Care

Some patients, particularly at the initial stage of CPAMS, had doubts about the quality of care they would receive at the pharmacies.^29^ For example, one person in New Zealand reported feeling like the computer was making the treatment decisions for them rather than the pharmacist.^29^

“The pharmacist does not manage my warfarin treatment—a computer programme does. I am not confident of the programme’s decisions.”^29^

A number of patients doubted the ability of pharmacists to manage their warfarin, with some believing that warfarin dose management may be well above their level of competence.^29^ Such considerations led to initial hesitancy in the uptake of CPAMS, especially among patients who had experienced previous adverse effects with warfarin.^27,29^

#### Resistance From GPs

Some CPAMS locations in New Zealand were unable to enroll their target number of patients because GPs withdrew or withheld their support.^27^ The pharmacists noted that some GPs would have found the program more acceptable if they had been approached via a GP organization rather than by the pharmacists directly.^27^ The GPs’ reluctance sometimes stemmed from their concern about who was ultimately responsible for the patients if things went wrong.^29^ Others were protective of their patients and did not see the need for pharmacists to do anticoagulation management,^36^ whereas some believed that the pharmacists were incapable of providing the service.^34^ Apathy toward a novel model of care was observed.^27,36^ There were also concerns about the warfarin doses recommended by the decision support system, with a number of GPs noting that the algorithm did not account for patient factors, such as noncompliance.^27^

#### Funding

In New Zealand, a number of pharmacists believed that the funding model for CPAMS, which was based on the number of patients enrolled at each pharmacy per month rather than the number of consults that patients required, was suboptimal.^34^ They noted that this costing model did not factor in the needs of complicated patients who may require additional consultations.^34^ Similarly, pharmacists in the Nova Scotia program noted that the monthly fee allocated for each patient was sufficient for the standard stable patient who was tested every 28 days but not for unstable patients with more frequent testing needs.^26^

Patients who had been receiving free GP and laboratory services had varying opinions about paying for CPAMS. Whereas some reported that they would happily pay for the service, others said that they would rather return to GP-led care if payment is required for participating in CPAMS after the pilot was over.^27^

“I would go back to lab if I did have to pay a fee for pharmacy.”^27^

Other patients said that they would struggle to pay for CPAMS but felt that the service was worth paying for.^27^ In Nova Scotia, for example, the majority of patients could not afford to pay the full costs of service, which was 54 CAD per month,^26^ whereas some patients in the Sydney program suggested a fee range from $10 to $20 per visit.^36^ In the Sydney program, the pharmacists believed that the level of remuneration did not reflect the time and effort required by them and the GPs.^36^

#### Capping

The CPAMS program in New Zealand capped the number of patients enrolled per pharmacy.^34^ This resulted in some patients, who wanted the service but could not access it, paying out of pocket for the service, whereas some others were placed on a waitlist. Additionally, there was a cap on the number of pharmacies providing CPAMS.^34^ Details regarding the extent of the capping were not provided in any of the New Zealand studies.

#### Organizational Limits

The amount of time spent on consultations at the beginning of CPAMS in New Zealand was initially high because all the patients were on a weekly INR testing regimen.^27^ Pharmacies had to juggle the staffing demands of CPAMS with meeting other service demands.^34^ For some pharmacies, the uncertainty around the amount of time to allocate to CPAMS at the expense of other services was a hindrance to uptake.^34^

“I think that you need to have a decent amount of staff in the pharmacy to be able to do it. I mean, you are taking up a pharmacist’s variable amount of time, sometimes it can be quick and easy, but other times sorting things out will take a bit longer. Not every pharmacy can do it.”^34^ (p. 260)

Furthermore, some pharmacies did not have the layout or staff that permits the implementation of CPAMS.^34,36^ One of the main challenges observed in the Nova Scotia program was the nonintegration of patients’ INR test results with the electronic health systems in the province. There were also challenges in communicating patients’ warfarin treatment to their GPs.^26^

## Discussion

This systematic review describes CPAMS implementation in 6 jurisdictions across 4 different countries. Although barriers, such as funding, capacity issues, and resistance by some GPs, were identified, CPAMS was shown to be convenient, accessible, efficient, and scalable. However, very few jurisdictions have fully adopted the program. The utilization of a decision support system for CPAMS also offered unique advantages, such as the calculation of recommended warfarin doses, appointment setting, calculation of the time in therapeutic range for each patient, and the mean time in therapeutic range for each pharmacy.^15^ It also tracks compliance with appointments, test frequency, and adverse events. Patient information can be easily retrieved, thereby ensuring efficient patient audit.^15^

A limitation of this systematic review is the reliance on a limited number of studies; thus, findings were mostly seen through the lens of a few researchers and a few jurisdictions. Second, we did not conduct quality assessment of the studies included in the review because the study types were varied and consisted mainly of reports.

## Relevance to Patient Care and Clinical Practice

CPAMS have been shown to improve safety outcomes in patients receiving warfarin therapy.^37,38^ However, the implementation of this model of AMS has not been widely adopted. This is the first systematic review evaluating the factors affecting CPAMS implementation across multiple jurisdictions. The barriers, facilitators, and implementation considerations identified in this review will inform health policy makers on how to implement and improve CPAMS for patients and health care practitioners.

## Conclusion and Relevance

CPAMS has been implemented in 6 jurisdictions across 4 countries, with reported benefits and challenges. Some of the benefits of CPAMS included convenience, increased collaboration between doctors and pharmacists, ability to scale, improved clinical outcomes, and increased efficiency. However, barriers to CPAMS were also identified, including lack of funding, resistance from GPs, organizational limits, and perceived poor quality of care by the patients. The programs were structurally similar in most jurisdictions, with minor variations in implementation. New anticoagulation management programs should consider adapting existing frameworks to local needs.

## Supplemental Material

sj-pdf-1-aop-10.1177_10600280211045075 – Supplemental material for A Qualitative Systematic Review of Facilitators of and Barriers to Community Pharmacists–Led Anticoagulation Management Service Click here for additional data file. Supplemental material, sj-pdf-1-aop-10.1177_10600280211045075 for A Qualitative Systematic Review of Facilitators of and Barriers to Community Pharmacists–Led Anticoagulation Management Service by Oluwaseun Egunsola, Joyce W. Li, Liza Mastikhina, Oluwasefunmi Akeju, Laura E. Dowsett and Fiona Clement in Annals of Pharmacotherapy

## Appendix

### Search Strategy

#### CINAHL CPAM Search

December 31, 2020

1. ( (MH "Anticoagulants") OR (MH "Warfarin") ) OR TI ( (adoisine or aldocumar or anticoagula* or anti-coagula* or antrombin or athrombin* or befarin or carfin or circuvit or coumadin* or coumafene or coumaphene or dagonal or farin or jantoven or kumatox or maforan or marevan or orfarin or panwarfarin or panwarfin or prothromadin or simarc-2 or sofarin or tedicumar or thrombin inhibitor* or tintorane or uniwarfin or waran or warfar or warfarin* or warfant or warnerin) ) OR AB ( (adoisine or aldocumar or anticoagula* or anti-coagula* or antrombin or athrombin* or befarin or carfin or circuvit or coumadin* or coumafene or coumaphene or dagonal or farin or jantoven or kumatox or maforan or marevan or orfarin or panwarfarin or panwarfin or prothromadin or simarc-2 or sofarin or tedicumar or thrombin inhibitor* or tintorane or uniwarfin or waran or warfar or warfarin* or warfant or warnerin) )
2. (MH "Pharmacy, Retail")
3. ( (MH "Pharmacy Service") OR (MH "Pharmacists") OR (MH "Pharmacy Technicians") ) OR TI ( (chemist or chemists or druggist* or pharmaceutical service* or pharmacist* or pharmacy or pharmacies) ) OR AB ( (chemist or chemists or druggist* or pharmaceutical service* or pharmacist* or pharmacy or pharmacies) )
4. (MH "Community Health Services") OR TI ( (community or communities or local or retail) ) OR AB ( (community or communities or local or retail) )
5. 3 and 4
6. TI ( (apothecaries or apothecary or chemist* shop* or drugstore* or drug store*) ) OR AB ( (apothecaries or apothecary or chemist* shop* or drugstore* or drug store*) )
7. 2 or 5 or 6
8. 1 and 7

#### MEDLINE

1. anticoagulants/ or warfarin/
2. (adoisine or aldocumar or anticoagula* or anti-coagula* or antrombin or athrombin* or befarin or carfin or circuvit or coumadin* or coumafene or coumaphene or dagonal or farin or jantoven or kumatox or maforan or marevan or orfarin or panwarfarin or panwarfin or prothromadin or simarc-2 or sofarin or tedicumar or thrombin inhibitor* or tintorane or uniwarfin or waran or warfar or warfarin* or warfant or warnerin).tw,kf.
3. 1 or 2
4. Community Pharmacy Services/
5. Pharmacies/
6. Pharmacists/
7. Pharmaceutical Services/
8. (chemist or chemists or druggist* or pharmaceutical service* or pharmacist* or pharmacy or pharmacies).tw,kf.
9. 5 or 6 or 7 or 8
10. (community or communities or local or retail).tw,kf.
11. 9 and 10
12. (apothecaries or apothecary or chemist* shop* or drugstore* or drug store*).tw,kf.
13. 4 or 11 or 12
14. 3 and 13
15. animals/ not humans/
16. 14 not 15

#### EMBASE

1. warfarin/
2. (adoisine or aldocumar or anticoagula* or anti-coagula* or antrombin or athrombin* or befarin or carfin or circuvit or coumadin* or coumafene or coumaphene or dagonal or farin or jantoven or kumatox or maforan or marevan or orfarin or panwarfarin or panwarfin or prothromadin or simarc-2 or sofarin or tedicumar or thrombin inhibitor* or tintorane or uniwarfin or waran or warfar or warfarin* or warfant or warnerin).tw,kw.
3. 1 or 2 or 3
4. "pharmacy (shop)"/
5. community pharmacist/
6. ((community or communities or local or retail) adj5 (chemist or chemists or druggist* or pharmacist* or pharmacy or pharmacies)).tw,kw.
7. (apothecaries or apothecary or chemist* shop* or drug store* or drugstore*).tw,kw.
8. 5 or 6 or 7 or 8
9. 4 and 9
10. animals/ not human/
11. 10 not 11

#### Cochrane Database of Systematic Reviews and DARE

1. (adoisine or aldocumar or anticoagula* or anti-coagula* or antrombin or athrombin* or befarin or carfin or circuvit or coumadin* or coumafene or coumaphene or dagonal or farin or jantoven or kumatox or maforan or marevan or orfarin or panwarfarin or panwarfin or prothromadin or simarc-2 or sofarin or tedicumar or thrombin inhibitor* or tintorane or uniwarfin or waran or warfar or warfarin* or warfant or warnerin).tw.
2. (chemist or chemists or druggist* or pharmaceutical service* or pharmacist* or pharmacy or pharmacies).tw.
3. (community or communities or local or retail).tw.
4. 2 and 3
5. (apothecaries or apothecary or chemist* shop* or drugstore* or drug store*).tw.
6. 4 or 5
7. 1 and 6

#### Cochrane Central Register of Controlled Trials

1. anticoagulants/ or warfarin/
2. (adoisine or aldocumar or anticoagula* or anti-coagula* or antrombin or athrombin* or befarin or carfin or circuvit or coumadin* or coumafene or coumaphene or dagonal or farin or jantoven or kumatox or maforan or marevan or orfarin or panwarfarin or panwarfin or prothromadin or simarc-2 or sofarin or tedicumar or thrombin inhibitor* or tintorane or uniwarfin or waran or warfar or warfarin* or warfant or warnerin).tw.
3. 1 or 2
4. Community Pharmacy Services/
5. Pharmacies/
6. Pharmacists/
7. Pharmaceutical Services/
8. (chemist or chemists or druggist* or pharmaceutical service* or pharmacist* or pharmacy or pharmacies).tw.
9. 5 or 6 or 7 or 8
10. (community or communities or local or retail).tw.
11. 9 and 10
12. (apothecaries or apothecary or chemist* shop* or drugstore* or drug store*).tw.
13. 4 or 11 or 12
14. 3 and 13
15. animals/ not humans/
16. 14 not 15
