# Supplementary figures and images for: A Qualitative Systematic Review of Facilitators of and Barriers to Community Pharmacists–Led Anticoagulation Management Service
Source: Ann Pharmacother. 2021 Sep 11;56(6):704–15. doi: 10.1177/10600280211045075 (PMC9008548; doi:10.1177/10600280211045075)

Supplementary Figure 1: CPAMS program structure in Nova Scotia

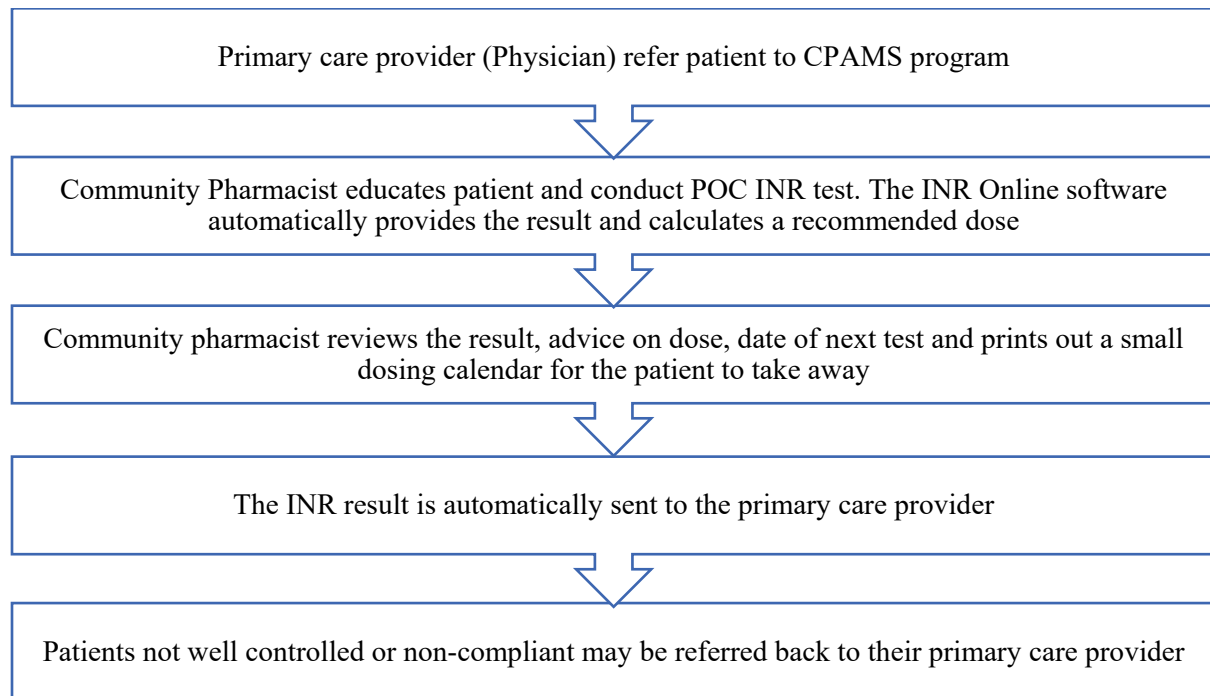

Supplement: sj-pdf-1-aop-10.1177_10600280211045075 – Supplemental material for A Qualitative Systematic Review of Facilitators of and Barriers to Community Pharmacists–Led Anticoagulation Management Service [file sj-pdf-1-aop-10.1177_10600280211045075.pdf]
